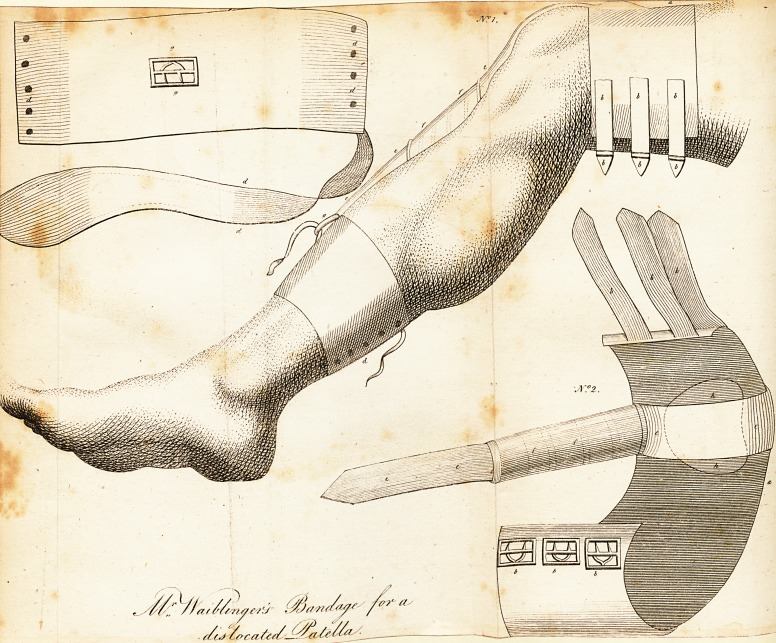# Case of William Walton, of Pudsey, a Young Man, 18 Years of Age

**Published:** 1800-10

**Authors:** I. Waiblinger

**Affiliations:** Fulneck, near Leeds, Member of the Royal College of Surgeons in London


### Medical and Phyfical Journal.

VOL. IV.] OCTOBER, 1800. [NO. XX.

### *Case of William Walton, of Pudfey, a young Man,* 18 *Years of Age; by* I. Waiblinger, of Fulneck, near Leeds, Member of the Royal College of Surgeons in London.

[ With an Engraving. ]

To the Editors of the Medical and Physical Journal.

Gentlemen,

About a year and a half ago, as the fubject of the following cafe was diverting himfelf with fome of his companions at the rural diverfion of hop, fpring, and leap, he fell fuddenly backwards; he felt no pain, but found himfelf unable to rife; on being raifed up by his companions, he found he could not ufe his right leg at all, though he made many efforts. From the circumftance of his feeling no pain, he had no idea that he had received any ferious injury. His companions, as foon as a horfe was procured, brought him to my houfe; on examining the knee, I found the *patella* was not in its place, but drawn up about three inches above the joint. The nature of the accident was now very obvious; the ftrong ligament which confines the *patella* to the tuberofity at the upper end of the *tibia*, was completely torn through, and the action of the ftrong extenfor mufcles inferted into the upper part of the *patella*, conftantly drew it upwards. The indication then plainly pointed out, was to overcome the action of the mufcles inferted into the *patella*. As I knew of no bandage adequate to this purpofe, and as I expected fome inflammation and tenfion would come on, which would for the prefent prevent any tight bandage being ufed, I applied a common roller round the thigh, rather as a placebo, than from any expectations I formed of its ufe; and a lotion of the *aqua lythargyr. acetat*, *&c.* to be frequently applied.

With the view of attaining the object of overcoming the action of the extenfor mufcles, and bringing the *patella* as near as poffible to its proper fituation, I invented the bandage of which I herewith fend a drawing and defcription; it has this recommendation, that it is not a mere fpeculative idea, but its utility is eftablifhed by the complete fuccefs attending its ufe in the above cafe. The young man is now perfectly well; the *patella* is in its proper place, and he is able to ufe his limb as well as ever he did in his life. I believe accidents exactly fimilar, are but rare; but I am perfuaded, that the bandage may be ufed with equal fuccefs in fractures of the *patella.* Admitting the difficulty of bringing the fractured parts of the bone into fuch clofe contact, as to form an offeous union; yet, furely, the nearer they are brought into contact, the lefs time will be required to form a ligamentous union. There are fome little improvements which may be added as circumftances require; but as they will not alter the principle of the bandage, I fhall not enter into them at prefent.—If the cafe related be found deferving of a place in your Repofitory, I fhall continue to furnifh you occafionally with fome *practical obfervations.* I am,

Your's, &c.

I. W.

No. 1. The bandage as applied.—No. 2. The fame in profile.

*a*, A leather ftrap, three inches and a half in breadth, lined with foft wafh leather, and fufficiently long to go round the limb above the knee, as is reprefented in No. 1; is faftened with three fmall buckles and correfponding ftraps, as at *b.*

*c*, A ftrap of the fame kind, faftened round the leg below the calf, the prominence of the gaftrocnemic mufcles preventing its being drawn upwards, faftened or laced tight with a leather thong on the underfide, as at *d*; and being fo far from the ftrap *a*, gives room for the elaftic fpiral fpring to act, which is contained in the ftrap *e.*

*e*, A ftrap thirteen inches long, firmly faftened to the broad ftrap *a*, and contains an elaftic wire fpring in the middle part of it, as at *f*, which fpring may be made to act at pleafure, by tightening or flackening the ftrap at the buckle *g*, which is inferted into the upper part of the ftrap *c*.

Under the edge of the ftrap *a*, is a hard fubftance faftened in, properly ftuffed, refembling in fize the patella, is rather concave at the lower edge, that it may more eafily hitch on to the convexity of the patella, as is fhewn in the drawing at *h*.

N. B. A pad of wool or tow, was worn under the ftrap *e*, where it croffes the joint, to prevent excoriation.

## Figures and Tables

**No. 1. No. 2. f1:**